# Pulmonary Lymphangitis Poses a Major Challenge for Radiologists in an Oncological Setting during the COVID-19 Pandemic

**DOI:** 10.3390/jpm12040624

**Published:** 2022-04-12

**Authors:** Roberta Fusco, Igino Simonetti, Stefania Ianniello, Alberta Villanacci, Francesca Grassi, Federica Dell’Aversana, Roberta Grassi, Diletta Cozzi, Eleonora Bicci, Pierpaolo Palumbo, Alessandra Borgheresi, Andrea Giovagnoni, Vittorio Miele, Antonio Barile, Vincenza Granata

**Affiliations:** 1 Medical Oncology Division, Igea SpA, 80013 Napoli, Italy; r.fusco@igeamedical.com; 2 Division of Radiology, Istituto Nazionale Tumori IRCCS Fondazione Pascale—IRCCS di Napoli, 80131 Naples, Italy; igino.simonetti@istitutotumori.na.it; 3 Diagnostica per Immagini nelle Malattie Infettive INMI Spallanzani IRCCS, 00161 Rome, Italy; stefania.ianniello@inmi.it (S.I.); alberta.villanacci@inmi.it (A.V.); 4 Division of Radiology, Università degli Studi della Campania Luigi Vanvitelli, 80127 Naples, Italy; francescagrassi1996@gmail.com (F.G.); federica.dellaversana@studenti.unicampania.it (F.D.); grassi.roberta89@gmail.com (R.G.); 5 Italian Society of Medical and Interventional Radiology (SIRM), SIRM Foundation, Via della Signora 2, 20122 Milan, Italy; cozzid@aou-careggi.toscana.it (D.C.); eleonora.bicci92@gmail.com (E.B.); alessandra.borgheresi@gmail.com (A.B.); a.giovagnoni@univpm.it (A.G.); vmiele@sirm.org (V.M.); 6 Department of Radiology, Azienda Ospedaliero-Universitaria Careggi, 50134 Florence, Italy; 7 Abruzzo Health Unit 1, Department of Diagnostic Imaging, Area of Cardiovascular and Interventional Imaging, 67100 L’Aquila, Italy; palumbopierpaolo89@gmail.com; 8 Department of Clinical, Special and Dental Sciences, Marche Polytechnic University, 60126 Ancona, Italy; 9 Department of Applied Clinical Science and Biotechnology, University of L’Aquila, Via Vetoio 1, 67100 L’Aquila, Italy; antonio.barile@univaq.it

**Keywords:** pulmonary lymphangitis, COVID 19, vaccination, computed tomography

## Abstract

Due to the increasing number of COVID-19-infected and vaccinated individuals, radiologists continue to see patients with COVID-19 pneumonitis and recall pneumonitis, which could result in additional workups and false-positive results. Moreover, cancer patients undergoing immunotherapy may show therapy-related pneumonitis during imaging management. This is otherwise known as immune checkpoint inhibitor-related pneumonitis. Following on from this background, radiologists should seek to know their patients’ COVID-19 infection and vaccination history. Knowing the imaging features related to COVID-19 infection and vaccination is critical to avoiding misleading results and alarmism in patients and clinicians.

## 1. Background

The current coronavirus disease 2019 (COVID-19) pandemic caused by severe acute respiratory syndrome coronavirus 2 (SARS-CoV-2) has produced a worldwide public health threat with millions of people at risk [1,2,3,4,5,6,7,8,9,10]. Globally, as of 5:18 PM CET on 4 March 2022, there were 440,807,756 confirmed cases of COVID-19, including 5,978,096 deaths, reported to the World Health Organization (WHO). In Italy, from 3 January 2020 at 5:18 p.m. CET to 4 March 2022, 12,910,506 confirmed cases of COVID-19 with 155,399 deaths have been registered, reported to the WHO [11].

The symptoms of COVID-19 infection are different from patient to patient, with the most common including fever, fatigue, cough, anorexia, and shortness of breath during different phases of this disease [12,13,14]. Additionally, less common symptoms such as a sore throat, headache, confusion, and chest tightness have been also observed [15,16], as well as minor gastrointestinal complications such as nausea, vomiting, and diarrhoea [17]. However, there have been patients not yet symptomatic (pre-symptomatic) and there have been patients without symptoms typical of the COVID-19 disease (asymptomatic), as revealed in several reports [18,19,20]. Several patients with COVID-19 infection were asymptomatic throughout the infection period [21,22]. In these asymptomatic patients the diagnosis was often accidental, as they performed radiological examinations for another cause or during the follow-up periods when dealing with cancer patients [23,24,25,26,27,28,29,30,31,32].

In addition, it has been critical to develop a vaccine as soon as possible to prevent SARS-CoV-2 infection in order to safeguard persons who are at a high risk of complications. Globally, on 27 February 2022, a total of 10,585,766,316 vaccine doses had been administered [11]. The Italian government employed the subsequent vaccination policy: two vaccine doses, with a booster dose 5–6 months after the end of the vaccination cycle, in patients who have not been infected. A policy for assessing vaccine adverse events is founded on the collaboration of local and national health structures, assisted by the Italian Medicines Agency (AIFA) [33]. Since the inception of vaccine inoculation, several adverse events have been reported and shared world-wide, with different findings reported by imaging studies [34,35,36,37,38,39,40,41,42,43,44]. While COVID-19-vaccine-related lymphadenopathy (LAP) has been gradually reported [34], few studies have reported lymphangitis after vaccination from COVID-19, and in several patients, radiation recall pneumonitis has been reported [45,46,47,48,49,50,51,52,53,54,55].

Since during the imaging management of oncological patients, the lung remains as the site of metastases, with different findings, including lymphangitis pattern [56,57,58,59,60,61], as well as the site of adverse events such as pneumonitis related to such therapies as Immune Checkpoint Inhibitor-Related Pneumonitis [62,63,64,65,66,67,68,69,70], it is clear that radiologists should know of previous COVID-19 infection or vaccination. The knowledge of these features related to COVID-19 infection or vaccination is critical in order to not presenting misleading results in patients and clinicians by determining disease progression or an adverse treatment effect.

This narrative review aims to evaluate the different patterns of pulmonary lymphangitis to improve the knowledge.

### Pulmonary Lymphangitis Carcinomatosa

**Figure 1:**
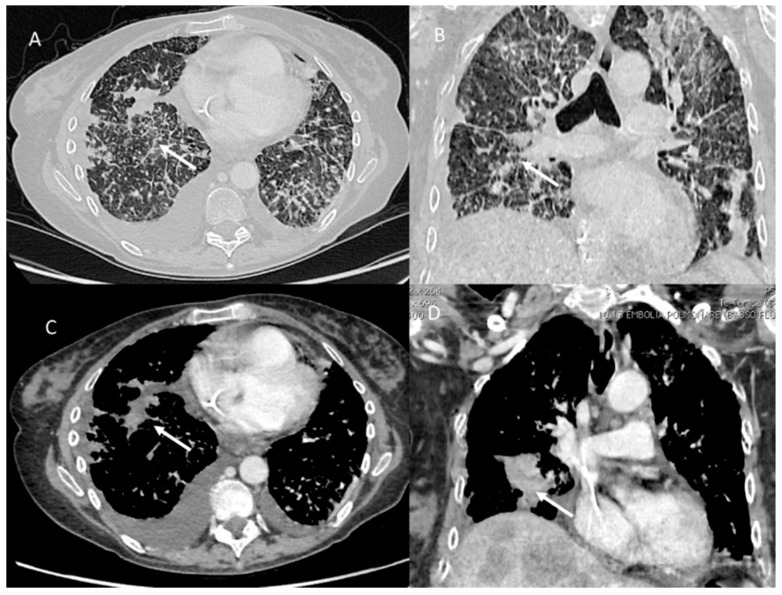
PLC in pancreatic cancer patient. CT (axial (**A**,**C**) and coronal (**B**,**D**)) shows comprise diffuse intrapulmonary infiltrates (arrows) with irregularly interlobular septal thickening, nodular thickening and pleural effusions.

Pulmonary lymphangitis carcinomatosa (PLC) is an unusual appearance of metastatic lung disease in which advanced cancers spread through pulmonary lymphatic vessels (Figure 1).

The difficult outflow of lymph from the lungs causes accumulation of interstitial fluid and disturbances in the diffusion of oxygen, which can cause respiratory dysfunction.

Imaging findings appear later than symptoms, and dyspnoea, representing the most frequent symptom, is habitually more severe than radiological findings [56,57,58]. The most common primary cancers that coexist with PLC are breast (17.3%), lung (10.8%) and stomach (10.8%) cancers [56]. According to Bruce et al. [70], the prevalence of PLC ranges from 6% to 8% in patients with malignant disease. Kazawa et al. [71] showed that the prevalence of PLC-related small-cell lung cancer was recognized in 8.8%. In patients with cancer and pulmonary embolism, the prevalence of PLC was 4.5% [72,73,74,75,76,77].

There are several hypotheses regarding the metastatic tumour spread, mainly limited to the lymphatics, although the precise process is still uncertain. The tumour could spread through the haematogenous route, causing obliterated endarteritis and then penetrating the vascular endothelium to reach the perivascular lymphatics, to remain stationed there. Direct entry into lymphatic circulation is possible when nearby lymph vessels are involved. Trans-diaphragmatic diffusion has also been suggested to clarify PLC due to abdominal cancers. Local obstruction and fluid accumulation follow soon after the malignant cells are trapped in the lymphatic vessels. This is followed by thickening of the bronchovascular bundles and alveolar septa due to tissue oedema and nodular thickening, suggesting local tumour cell growth.

Imaging has low specificity for PLC detection because images are often normal in initial phases. However, imaging is often performed to rule out other causes [57,78,79,80,81,82,83,84,85]. A chest radiograph (chest X-ray) may be the first approach and an association of several radiological findings could favour the diagnosis in about half the cases. However, in 30 to 50% of patients, especially in the initial phases, the X-ray may be normal [56,57]. Therefore, computed tomography (CT) and especially high-resolution CT (HRCT) are recommended techniques for studying patients with suspected PLC [55,56,57,86,87,88,89,90,91,92,93]. The CT results are similar to other interstitial lung diseases, so they have low diagnostic accuracy in differentiating and detecting PLC [56,57]. The usual CT findings comprise nodular or diffuse intrapulmonary infiltrates, irregularly interlobular septal thickening, smooth (early stage) or nodular thickening (late development), hilar and mediastinal lymphadenopathy, ground-glass opacity due to interstitial oedema or parenchymal extension of tumours, and pleural effusions. These features could be on one or both sides, focal or diffuse, or symmetrical or asymmetrical. Smooth or irregularly thickened interlobular septae are more conducive to PLC than to tumour embolism. Although nodular thickening of the septae is thought to differentiate PLC from other interstitial lung patterns, some conditions such as sarcoidosis or asbestosis may mimic it [57]. Another distinguishing feature of PLC is the preservation of the general and lobular architecture of the lung. [56,57].

## 2. Immune Checkpoint Inhibitor-Related Pneumonitis

Anti-programmed death-1 (anti-PD-1) and programmed anti-death ligand 1 (anti-PD-L1) monoclonal antibodies (mAb) for patients with multiple cancers are licensed treatments, including nivolumab and pem-brolizumab for melanoma and non-small-cell lung cancer (NSCLC), nivolumab for renal cell carcinoma and Hodgkin’s lymphoma, atezolizumab for bladder cancer, and nivolumab plus ipilimumab for melanoma [94,95,96,97,98,99,100,101,102,103]. A major feature of anti-PD-1/PD-L1 mAbs is their mild toxicity, but serious immune-related adverse events can occur. Pneumonia is an immune-related adverse event defined as focal or diffuse inflammation of the lung parenchyma, and its incidence in studies with anti-PD-1/PD-L1 mAbs ranges from 0% to 10% [104]. Drug-related pneumonia can also occur with chemotherapy (docetaxel [105], gemcitabine [106], bleomycin [107]), targeted therapy (epidermal growth factor receptor inhibitors [108,109], mammalian target of rapamycin inhibitors [110]), and radiotherapy [111,112].

Compared to conventional chemotherapy for pneumonia, these patients showed a greater susceptibility to the development of treatment-related pneumonia, with an increased risk of high-grade pneumonia. Previous studies on this pneumonia have shown that clinical, radiological and pathological characterization can facilitate early diagnosis to improve patient outcomes [113,114,115,116,117]. The aetiology and underlying mechanisms of anti-PD-1/PD-L1 mAb-associated pneumonia are unknown [63,118,119,120,121].

**Figure 2:**
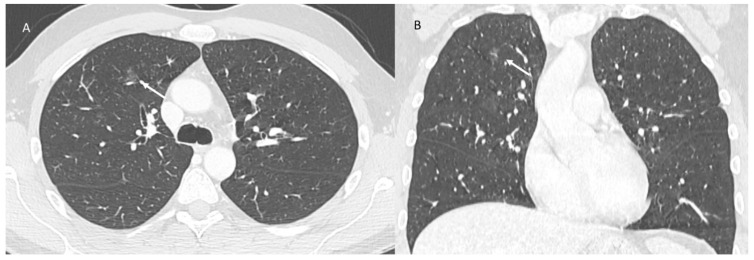
ICI-related pneumonitis. OP pattern on CT (axial: (**A**) and coronal: (**B**)): ground-glass and airspace opacities (arrows).

Immune checkpoint inhibitor (ICI)-related pneumonia could cause significant morbidity, with possible discontinuation of therapy and possible mortality [118]. The time to onset of pneumonia ranges from 9 days to over 19 months after the initiation of therapy, with a median time of 2.8 months [118]. Imaging plays a crucial role in this effect detection. Although X-ray may be an initial tool, CT is able to detect all subtle changes in pneumonitis and help to differentiate among subtypes, as described by Delaunay et al. [122]. Investigations described 64 cases of pneumonia with the following CT patterns: (a) organized pneumonia (OP) (23%), (b) hypersensitivity pneumonitis (HP) (16%), (c) non-specific interstitial pneumonia (NSIP) (8%) and (d) bronchiolitis (6%). Some patients were diagnosed with concomitant patterns and a distinctive pattern was not identified in 36% of cases [122]. OP’s pattern usually shows bilateral peribronchovascular and subpleural ground-glass and airspace opacities, with mid- to lower-lung predominance (Figure 2).

In addition, an inverted halo or atoll sign is detected. Pulmonary nodules, usually with peribroncovascular distribution and generally smaller (<10 mm) nodules, may also be detected. However, in some cases, the nodules may be nodular and massive with pointed edges, mimicking findings of malignancy [118,122]. These features should be distinguished from progression of malignancy (concurrent worsening of disease in other areas) and infection (clinical history, laboratory findings, response to appropriate therapy).

The NSIP pattern commonly manifests as ground glass and lattice opacities predominantly in the lower lobe. Airspace disease is temporally homogeneous and relatively symmetrical, with uncommon consolidation opacities, allowing NSIP patterns to be distinguished from OP patterns. Subpleural sparing of the posterior and inferior lobes has also been described as a specific feature. NSIP pattern should be distinguished from NSIP associated with autoimmune or connective tissue disease (appropriate medical history and condition-specific markers, no temporal relationship to immunotherapy course) and infection (clinical history, laboratory findings, response to appropriate therapy).

The HP pattern is associated with lower-grade symptoms. CT findings include diffuse or predominant ground-glass centrilobular nodules in the upper lobe, which may be related to air entrapment. This pattern should be distinguished from exposure-related HP (exposure and occupational history, no temporal relationship to immunotherapy course), and from respiratory and follicular bronchiolitis (smoking history or underlying connective tissue and/or autoimmune disease history) and atypical infection (clinical history, laboratory findings, response to appropriate therapy). 

**Figure 3:**
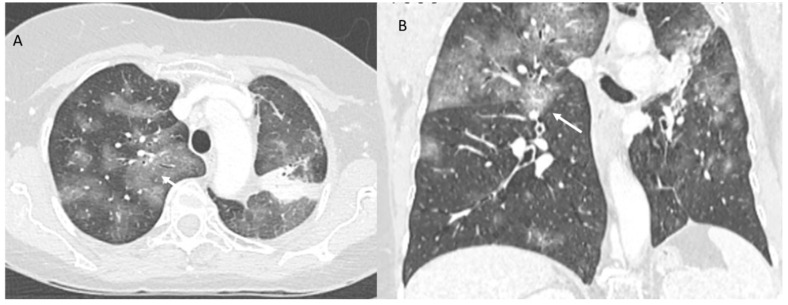
ICI-related pneumonitis. AIP-ARDS pattern on CT (axial: (**A**) and coronal: (**B**)): diffuse ground-glass opacities involving a majority of the lungs (arrows), although areas of lobular sparing can be detected.

Acute interstitial pneumonia (AIP)–acute respiratory distress syndrome (ARDS) is not a model of ICI therapy-related pneumonia, although it is associated with a more severe clinical course and extensive pulmonary involvement with imaging. This pattern is characterized by geographic or diffuse ground-glass or consolidation opacities involving most, and sometimes all, of the lungs, although lobular sparing areas may be detected. There may also be a thickening of the interlobular septum and a “crazy pavement” pattern (Figure 3). The differential diagnosis is extensive and includes pulmonary oedema, haemorrhage, and infection. The findings of ARDS may also be due to extrapulmonary causes such as pancreatitis, sepsis and/or shock and transfusion reactions.

The bronchiolitis pattern appears as a region of centrilobular nodularity, often ten in a tree-in-bud pattern. Thickening of the adjacent bronchial wall is frequently observed, as well as focal ground-glass and consolidation opacity, although this should not be the main feature. This pattern should be distinguished from aspiration (dependent lungs, airway and oesophageal secretion) and infection (clinical history, laboratory findings, response to appropriate therapy).

### 2.1. COVID-19 Vaccine Radiation Recall

Radiation recall reaction (RRR) is an infrequent but well-known event to clinicians, characterized by a late-occurring acute inflammatory reaction that develops in confined areas corresponding to previously irradiated radiation therapy (RT) treatment fields. RRP has been known to be triggered by a number of chemotherapy agents [123,124,125,126,127,128,129,130,131,132], including, recently, even COVID-19 vaccines [45]. It occurs in a variety of tissues, the commonest being skin, which accounts for two-thirds of reported cases. It is usually mild and self-limiting when the trigger drug is stopped. Re-challenge with the drug does not necessarily cause reactivation of the reaction. 

**Figure 4:**
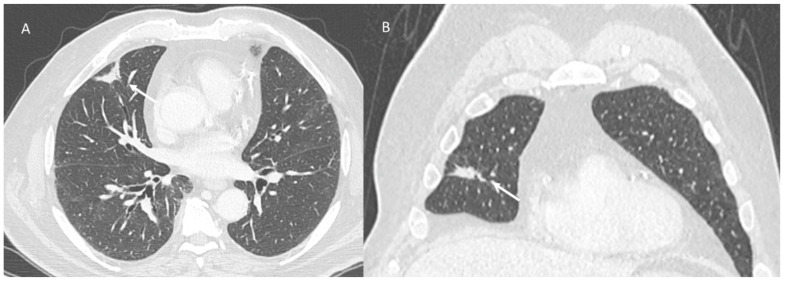
Radiation recall pneumonitis (CT scan axial: (**A**) and coronal: (**B**)) pattern includes consolidative opacities limited (arrows) to a prior radiation field.

This event has been reported within the lungs [52,118,133], determining radiation recall pneumonitis as acute inflammation within a previously irradiated area. The mechanism of the disease is unclear, but it seems to be related to an immune response. COVID-19 infection is known to cause immunological reactions, such as cytokine storm or multisystem inflammatory syndrome, in children [134,135,136,137]. Just like real infection, the developed vaccines are expected to induce an immune response. The inflammatory state created by the vaccine can favour the development of radiation recall. In fact, the few available papers on the topic suggest that COVID-19 vaccine can cause RRR, considering the time of vaccine administration and this event (Figure 4) [52,133]. The radioactive recall pneumonia model includes consolidated or ground-glass opacities. It should be suspected in all patients with previous radiotherapy with new airspace changes clearly demarcated from the adjacent lung. The main differential diagnosis is infection that does not respect boundaries and occurs outside of the previous radiation field. 

### 2.2. Pneumonitis COVID-19

Typical chest CT imaging findings for COVID-19 patients are ground-glass opacity with bilateral multifocal patches or consolidation with the interlobular septum and vascular thickening in peripheral areas of the lungs [134,135,136,137,138,139,140,141,142]. These manifestations may also be compatible with other viral pneumonias [143]. In this scenario, the current gold standard for diagnosing COVID-19 is based on a molecular reverse transcription polymerase chain reaction (RT-PCR) test, aimed at detecting virus RNA in respiratory samples such as nasopharynx swabs or bronchial aspirate [144].

**Figure 5:**
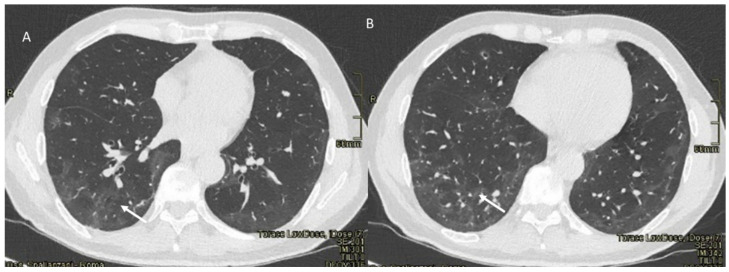
COVID-19 patient. CT (axial plane: (**A**,**B**)) shows early stage with GGO (arrow) as main finding.

**Figure 6:**
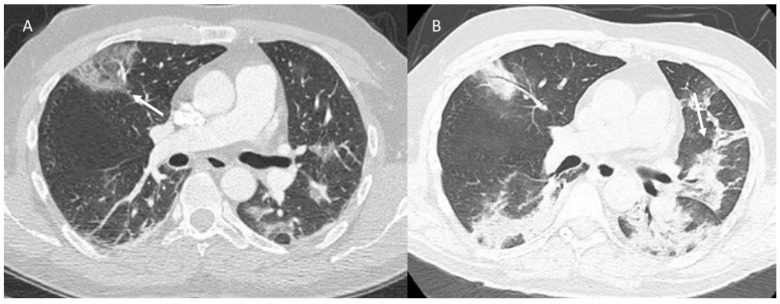
COVID-19 patient at peak stage. CT (axial plane: (**A**,**B**)) shows consolidation (arrow).

**Figure 7:**
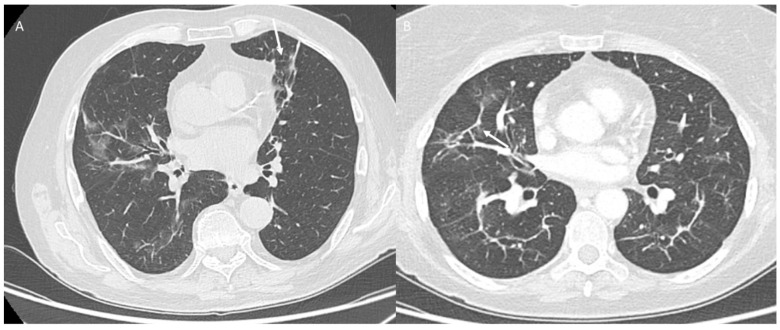
COVID-19 patient at late stage. CT (axial plane: (**A**,**B**)) shows fibrotic-like changes (arrow).

Compared to RT-PCR, the specificity of CT in detecting COVID-19 is lower, with an overall reported specificity of 46–80% [145,146,147]. This is due to the fact that the typical pattern of COVID-19 pneumonia shows a partial overlap with that of other lung diseases: ground-glass opacity (GGO), consolidation, crazy pavement, and enlargement of subsegmental vessels (diameter greater than 3 mm) in the GGO areas [148,149,150]. The temporal course of these anomalies was described by Pan et al., reporting four phases of the disease: initial stage (0–4 days after the onset of symptoms) with GGO as the main finding (Figure 5), progressive stage (5–8 days after onset of symptoms) with widespread GGO, mad pattern and consolidation, peak stage (9–13 days after onset of symptoms) with consolidation becoming more prevalent (Figure 6) and advanced stage (≥14 days after the onset of symptoms) with gradual absorption of anomalies (Figure 7) [151]. A recent study examined the performance of radiologists in differentiating COVID-19 from non-COVID-19 viral pneumonia, revealing an accuracy of between 60 and 83% [152].

COVID-19 pneumonia may be misdiagnosed as non-COVID-19 lung disease in the early and late stages, reflecting the fact that the typical early-stage pattern and the absorption of late features are commonly linked to signs of organizational pneumonia or signs of early fibrosis. These characteristics can be found in different conditions and, therefore, this aspect should be considered non-specific [148,149,150,151,152].

## 3. Discussion

Since the population continues to be infected or vaccinated in larger numbers, COVID-19 pneumonitis and RRR pneumonitis caused by COVID-19 vaccination will be increasingly seen by radiologists and could result in imaging test call-backs, additional workups, and false-positive results. In addition, considering oncological patients who could develop drug-related pneumonitis or a pattern of PLC as a progressive disease, it is clear as these conditions should be taken into account for a correct diagnosis. Therefore, a correct medical history is essential to ruling out the possibility of a recent infection as well as recent vaccine administration. 

RRR and SARS-CoV-2 interstitial pneumonia, so non-COVID-19-related pneumonia, show overlapping clinical features. In fact, the most common symptoms are dyspnoea and dry non-productive cough and fever. Additionally, chest CT findings are also very similar, as the radiological characteristics of COVID-19 and non-COVID-19-related pneumonia are GGO in the initial phase, patchy areas of consolidation in the peak phase and fibrotic changes in the dissipative phase [8,14]. 

Where the cause of the lung abnormalities is unclear, due to radiological CT findings being unspecific, multidisciplinary management would be correct to establish the main proper treatment. Although the management of oncological patients should consider the probability of malignant lung involvement with disease progression or adverse effects of specific therapies, it is clear that in pandemic conditions, several unspecific features may be related to an undiagnosed infection. Although the typical pattern of COVID-19 pneumonitis should be identified with high accuracy in symptomatic patients, this could be complicated if infection has been not diagnosed, especially in the post-infection phase. Therefore, we should consider several features, such as that the typical RRR pattern is usually strictly related to the target volume and to the dose distribution of the treatment plan, while during COVID-19 infection, the parenchymal involvement is not limited to a single lobe, although this is possible during the first phase. Additionally, PLC and ICI-related pneumonitis show diffuse parenchymal involvement. Additionally, in PLC, we find an irregularly interlobular septal thickening or nodular thickening, while in COVID-19 pneumonitis and RRR, septal thickening is more regular. These differences in chest CT patterns are the main factors that should help lead to a proper diagnosis (Table 1). However, distinguishing COVID-19 lung involvement or vaccine RRR from other lung pathologies such as cancer on chest CT may be straightforward, differentiation between COVID-19 and other pneumonias can be particularly troublesome for physicians because of the radiographic similarities, and this is particularly evident during the early or late phases of these pathologies. Inaccurate imaging interpretation makes it harder for patient management strategies to work efficiently. 

Interestingly, COVID-19 pneumonia and ICI-related pneumonitis have been suggested to share critical biological mechanisms, including the hyperactivation of immune cells associated with a significant increase in pro-inflammatory cytokines. Distinguishing between COVID-19 pneumonia and ICI-related pneumonitis is a diagnostic challenge. ICI-related pneumonitis might present with several patterns. These patterns seemingly overlap the CT features of COVID-19 pneumonia, possibly due to overlapped biological mechanisms. 

Recently, the progressive integration of radiomics approaches and artificial intelligence (AI)-based solutions could be of help. To date, the application of AI in medical imaging has improved the assessment and early diagnosis of neurodegenerative diseases and heart disease, with a particularly high impact on breast and lung cancer [153,154,155,156,157,158,159,160,161,162]. A cutting-edge research direction leverages deep learning (DL) and machine learning (ML) to understand COVID-19. AI could be used for the detection and quantification of COVID-19 disease from X-ray and CT images [163,164,165], enabling correct patient diagnosis and management. Deep learning (DL), a form of AI, has been successfully applied to chest CT imaging to distinguish COVID-19 pneumonia from community-acquired infections, as well as to provide qualitative and quantitative analyses for disease burden estimation and facilitating and expediting imaging interpretation [166]. However, deep learning algorithms based only on CT images cannot distinguish COVID-19 pneumonia from other lung interstitial diseases with overlapping CT features with high specificity; thus, adding clinical/laboratory findings to the algorithm can improve the diagnostic performance based on binary classification [167,168].

## 4. Conclusions

Knowing the chest imaging features related to COVID-19 infection or vaccine is critical to avoid misleading results in patients and clinicians and determine the idea of a disease progression or an adverse treatment effect. In this context, we should consider several features, as the typical RRR pattern is usually strictly related to the target volume and the dose distribution of the treatment plan. At the same time, during COVID-19 infection, parenchymal involvement is not limited to a single lobe, although this is possible during the first phase. Additionally, PLC and ICI-related pneumonitis show a diffuse parenchymal involvement. The PLC shows an irregularly interlobular septal thickening or nodular thickening, while septal thickening is common in COVID-19 pneumonitis and RRR. These differences in chest CT patterns are the main factors that should help for proper diagnosis. However, distinguishing COVID-19 lung involvement or vaccine RRR from other lung pathologies on chest CT may be straightforward, particularly during the early or late phases of these pathologies.

## Figures and Tables

**Table 1:** Lung involvement and CT pattern for pneumonia type.

Type of Pneumonia	Lung Involvement	CT-Patter
COVID-19 Pneumonia	Diffuse (related to the phase of disease)	ground-glass opacity, crazy-paving pattern, consolidative opacities, interlobular septal thickening (according to the phase of disease)
RRR-Related Vaccine	Target Area	Consolidative opacities
Pulmonary lymphangitis carcinomatosa	Diffuse (related to the phase of disease)	Irregularly interlobular septal thickening, smooth (early stage), or nodular thickening (late development), ground-glass opacity, pleural effusions.
ICI-Related Pneumonitis	Diffuse (related to the phase of disease)	ground-glass and reticular opacities, consolidative opacities, interlobular septal thickening, “crazy-paving” pattern

## Data Availability

Data are available at https://zenodo.org/record/6393020#.YkgKd-hBy3A (accessed on 21 January 2022).
